# Supplementary material for: Exploring taxonomic and functional microbiome of Hawaiian stream and spring irrigation water systems using Illumina and Oxford Nanopore sequencing platforms
Source: Front Microbiol. 2023 Feb 17;14:1039292. doi: 10.3389/fmicb.2023.1039292 (PMC9981659; doi:10.3389/fmicb.2023.1039292)
Supplement: Supplementary file 1 [file Table_1.DOCX]

**Table S1.** List of samples collected across the Oahu Islands for the study.

| **Sample*** | **Sample water type** | **Direction** |
| --- | --- | --- |
| R-F1-E | Field water (Taro) | East |
| R-S1-E | River stream | East |
| R-S2-W | River stream | North |
| R-F2-W | Field water (Taro) | North |
| S-S3-N | Spring water | North |
| S-F3-N | Field water (Taro) | North |
| R-S4-SE | River stream | South-East |
| R-F4-SE | Field water (Taro) | South-East |
| R-S5-SE | River stream | South-East |
| R-F5-SE | Field water (Taro) | South-East |
| T-S6-N | Tank water | North |
| R-S7-N | Stream water (Horticultural crops) | North |

*Irrigation source-sample water type#(ID)-direction

**Table S2.** List of samples sequenced using Illumina iSeq100. Assigned barcodes with different combinations of i5 and i7 adapters. Each sample was sequenced in two replicates.

| **Sample** | **Replicate 1** | **Replicate 2** |
| --- | --- | --- |
| R-F1-E | Barcode 1 (i517-i710 adapter) | Barcode 13 (i506-i710 adapter) |
| R-S1-E | Barcode 2 (i517-i705 adapter) | Barcode 14 (i506-i705 adapter) |
| R-S2-W | Barcode 3 (i517-i706 adapter) | Barcode 15 (i506-i706 adapter) |
| R-F2-W | Barcode 4 (i517-i707 adapter) | Barcode 16 (i506-i707 adapter) |
| S-S3-N | Barcode 5 (i517-i711 adapter) | Barcode 17 (i506-i711 adapter) |
| S-F3-N | Barcode 6 (i517-i714 adapter) | Barcode 18 (i506-i714 adapter) |
| R-S4-SE | Barcode 7 (i505-i710 adapter) | Barcode 19 (i503-i710 adapter) |
| R-F4-SE | Barcode 8 (i505-i705 adapter) | Barcode 20 (i503-i705 adapter) |
| R-S5-SE | Barcode 9 (i505-i706 adapter) | Barcode 21 (i503-i706 adapter) |
| R-F5-SE | Barcode 10 (i505-i707 adapter) | Barcode 22 (i503-i707 adapter) |
| T-S6-N | Barcode 11 (i505-i711 adapter) | Barcode 23 (i503-i711 adapter) |
| R-S7-N | Barcode 12 (i505-i714 adapter) | Barcode 24 (i503-i714 adapter) |

**Table S3.** Valid reads after quality filtration with calculated average read length generated using Illumina iSeq100 sequencer.

| **Sample** | **Barcode** | **Valid reads** | **Average length (bp)** | **Barcode** | **Valid reads** | **Average length (bp)** |
| --- | --- | --- | --- | --- | --- | --- |
| R-F1-E | 1 | 50,796 | 286.8 | 13 | 47,573 | 286.6 |
| R-S1-E | 2 | 47,032 | 282.9 | 14 | 48,790 | 282.9 |
| R-S2-W | 3 | 61,272 | 283.2 | 15 | 67,325 | 283.2 |
| R-F2-W | 4 | 28,288 | 283.6 | 16 | 31,661 | 283.5 |
| S-S3-N | 5 | 47,654 | 282.4 | 17 | - | - |
| S-F3-N | 6 | 74,525 | 285.9 | 18 | 63,230 | 285.8 |
| R-S4-SE | 7 | 31,574 | 284.0 | 19 | 28,530 | 284.1 |
| R-F4-SE | 8 | 22,274 | 284.3 | 20 | 26,908 | 284.3 |
| R-S5-SE | 9 | 43,658 | 282.0 | 21 | 46,548 | 282.1 |
| R-F5-SE | 10 | 35,595 | 284.1 | 22 | 44,228 | 284.1 |
| T-S6-N | 11 | 47,020 | 281.2 | 23 | 55,980 | 281.3 |
| R-S7-N | 12 | 33,495 | 282.6 | 24 | 33,187 | 282.7 |

**Table S4.** Statistical analysis of the identified phyla among all the samples was determined using one-way ANOVA (single factor) with the least significant difference (LSD) test at α=0.05.

| Phylum | | | Count | | Sum | | Average | Variance |  |  |  |
| --- | --- | --- | --- | --- | --- | --- | --- | --- | --- | --- | --- |
| Acidobacteria | | | 12 | | 8.0138 | | 0.667817 | 0.459307 |  |  |  |
| Actinobacteria | | | 12 | | 55.2168 | | 4.6014 | 54.25061 |  |  |  |
| Bacteroidetes | | | 12 | | 122.506 | | 10.20883 | 215.7226 |  |  |  |
| Chlorobi | | | 12 | | 7.0533 | | 0.587775 | 1.321251 |  |  |  |
| Chloroflexi | | | 12 | | 4.8577 | | 0.404808 | 0.120637 |  |  |  |
| Cyanobacteria | | | 12 | | 108.4629 | | 9.038575 | 348.6479 |  |  |  |
| Elusimicrobia | | | 12 | | 3.3087 | | 0.275725 | 0.114638 |  |  |  |
| Fibrobacteres | | | 12 | | 3.3986 | | 0.283217 | 0.087266 |  |  |  |
| Firmicutes | | | 12 | | 44.4733 | | 3.706108 | 39.62349 |  |  |  |
| JMYB36 | | | 12 | | 1.733 | | 0.144417 | 0.15651 |  |  |  |
| Kazan | | | 12 | | 4.4133 | | 0.367775 | 0.178326 |  |  |  |
| Omnitrophica_OP3 | | | 12 | | 3.5871 | | 0.298925 | 0.174675 |  |  |  |
| Parcubacteria_OD1 | | | 12 | | 48.4779 | | 4.039825 | 26.67713 |  |  |  |
| Peregrinibacteria | | | 12 | | 4.3685 | | 0.364042 | 0.116108 |  |  |  |
| Planctomycetes | | | 12 | | 11.6281 | | 0.969008 | 0.646273 |  |  |  |
| Proteobacteria* | | | 12 | | 702.3075 | | 58.52563 | 641.4002 |  |  |  |
| Saccharibacteria_TM7 | | | 12 | | 13.0376 | | 1.086467 | 1.051675 |  |  |  |
| Verrucomicrobia | | | 12 | | 36.3249 | | 3.027075 | 25.42415 |  |  |  |
| One way-ANOVA | | | | | | | | | | | |
| *Source of Variation* | | *SS* | *df* | | *MS* | | *F* | | *P-value* | | *F crit* |
| Between Groups | | 37621.02 | 17 | | 2213.001 | | 29.37238 | | 5.63E-45 | | 1.674587 |
| Within Groups | | 14917.9 | 198 | | 75.34293 | |  | |  | |  |
| Total | | 52538.92 | 215 | |  | |  | |  | |  |

Significant if difference in variance between two compared phyla was greater than calculated LSD value (6.988063).

*Statistically significant among all other phyla identified.

**Table S5.** Statistical analysis of identified genera among all the samples was determined using one-way ANOVA (single factor) with the least significant difference (LSD) test at α=0.05.

| Genus | Samples | Sum | Average | Variance |
| --- | --- | --- | --- | --- |
| AF236014_g | 12 | 26.4928 | 2.207733 | 13.93174 |
| *Acidibacter* | 12 | 6.1776 | 0.5148 | 0.976789 |
| *Acinetobacter* | 12 | 2.5366 | 0.211383 | 0.151579 |
| *Arcobacter* | 12 | 16.9884 | 1.4157 | 7.655345 |
| *Azonexus* | 12 | 1.8406 | 0.153383 | 0.104587 |
| Burkholderiaceae_uc | 12 | 2.7073 | 0.225608 | 0.119841 |
| Flavobacteriaceae_uc | 12 | 9.3295 | 0.777458 | 3.698323 |
| GU305779_g | 12 | 3.1472 | 0.262267 | 0.339311 |
| *Mycobacterium* | 12 | 6.2091 | 0.517425 | 1.301162 |
| PAC000128_g | 12 | 2.146 | 0.178833 | 0.0872 |
| *Prevotella* | 12 | 2.8556 | 0.237967 | 0.155507 |
| *Prochlorococcus* | 12 | 105.1676 | 8.763967 | 351.5253 |
| Rhodocyclaceae_uc | 12 | 2.0787 | 0.173225 | 0.114571 |
| *Roseomonas* | 12 | 2.3434 | 0.195283 | 0.136651 |
| *Sulfuricurvum* | 12 | 1.4322 | 0.11935 | 0.089258 |
| *Tabrizicola* | 12 | 1.5307 | 0.127558 | 0.093728 |
| FJ437985_g | 12 | 2.0832 | 0.1736 | 0.206132 |
| *Flavobacterium* | 12 | 62.903 | 5.241917 | 123.6531 |
| *Hyphomicrobium* | 12 | 3.1922 | 0.266017 | 0.07914 |
| JN087872_g | 12 | 3.8791 | 0.323258 | 0.518839 |
| *Planktophila* | 12 | 4.9744 | 0.414533 | 0.630999 |
| *Polynucleobacter* | 12 | 5.2169 | 0.434742 | 0.17564 |
| Sphingomonadaceae_uc | 12 | 5.6299 | 0.469158 | 0.239249 |
| AY532578_g | 12 | 6.8333 | 0.569442 | 0.854341 |
| *Bdellovibrio* | 12 | 4.3863 | 0.365525 | 0.130646 |
| *Cellvibrio* | 12 | 9.3697 | 0.780808 | 0.641694 |
| EU803579_g | 12 | 1.7869 | 0.148908 | 0.117079 |
| *Fluviicola* | 12 | 4.3954 | 0.366283 | 0.286912 |
| HQ343229_g | 12 | 2.384 | 0.198667 | 0.249013 |
| LBRP_g | 12 | 7.924 | 0.660333 | 0.883163 |
| LCRP_g | 12 | 2.3479 | 0.195658 | 0.094942 |
| *Nanopelagicus* | 12 | 10.4248 | 0.868733 | 4.533814 |
| Oxalobacteraceae_uc | 12 | 9.1001 | 0.758342 | 0.399625 |
| PAC000016_g | 12 | 6.8106 | 0.56755 | 0.320557 |
| Paenibacillaceae_uc | 12 | 11.7132 | 0.9761 | 11.27143 |
| *Paenibacillus* | 12 | 4.6467 | 0.387225 | 1.413813 |
| Pedobacter_g3 | 12 | 1.5937 | 0.132808 | 0.106075 |
| *Sediminibacterium* | 12 | 3.6725 | 0.306042 | 0.333551 |
| *Acidovorax* | 12 | 6.9365 | 0.578042 | 0.442103 |
| *Lampropedia* | 12 | 5.2395 | 0.436625 | 0.112086 |
| *Methylomonas* | 12 | 2.5097 | 0.209142 | 0.377205 |
| *Rhizobacter* | 12 | 7.9287 | 0.660725 | 0.293389 |
| *Saccharimonas* | 12 | 3.8207 | 0.318392 | 0.116723 |
| Selenomonadaceae_uc | 12 | 2.7298 | 0.227483 | 0.133856 |
| AF370880_g | 12 | 3.6005 | 0.300042 | 0.171381 |
| *Bacteroides* | 12 | 1.5219 | 0.126825 | 0.092735 |
| CP011215_f_uc | 12 | 17.8864 | 1.490533 | 3.116723 |
| Chthoniobacteraceae_uc | 12 | 3.8789 | 0.323242 | 0.620249 |
| Comamonadaceae_uc* | 12 | 217.7156 | 18.14297 | 164.9515 |
| Cytophagaceae_uc | 12 | 5.7242 | 0.477017 | 0.859744 |
| GQ387490_g | 12 | 14.3576 | 1.196467 | 10.34723 |
| HQ827934_g | 12 | 4.6018 | 0.383483 | 0.444987 |
| Methylophilaceae_uc | 12 | 5.9172 | 0.4931 | 0.207527 |
| *Pseudarcicella* | 12 | 8.8489 | 0.737408 | 1.999995 |
| *Rheinheimera* | 12 | 11.6233 | 0.968608 | 2.543325 |
| *Sphingomonas* | 12 | 4.8219 | 0.401825 | 0.143136 |

| One way-ANOVA | | | | | | |
| --- | --- | --- | --- | --- | --- | --- |
| *Source of Variation* | *SS* | *df* | *MS* | *F* | *P-value* | *F crit* |
| Between Groups | 4729.362 | 55 | 85.9884 | 6.738576 | 8.26E-36 | 1.354723 |
| Within Groups | 7860.541 | 616 | 12.76062 |  |  |  |
| Total | 12589.9 | 671 |  |  |  |  |

Significant if difference in variance between two compared genera was greater than calculated LSD value (2.863933). *Statistically significant among all other identified genera.

**Table S6.** Short length 16S rRNA reads classified to genera level, accounting for relative abundance >1%, <1% and remains unclassified analyzed using EzBioCloud.

| Samples | Genus | | Species | |
| --- | --- | --- | --- | --- |
|  | ETC (<1%) | Unclassified | ETC (<1%) | Unclassified |
| R-F1-E | 22.3221 | 6.851 | 23.1975 | 50.9293 |
| R-S1-E | 45.7396 | 10.4831 | 37.9949 | 55.8858 |
| R-S2-W | 60.9992 | 9.4146 | 53.1965 | 43.7102 |
| R-F2-W | 44.5452 | 11.1655 | 33.3572 | 55.2932 |
| S-S3-N | 34.3494 | 6.4156 | 25.6981 | 33.1059 |
| S-F3-N | 19.3498 | 7.8432 | 27.5119 | 33.3124 |
| R-S4-SE | 44.1636 | 21.5543 | 41.874 | 52.4737 |
| R-F4-SE | 34.7894 | 19.0581 | 34.4392 | 62.2295 |
| R-S5-SE | 61.8705 | 11.0308 | 54.319 | 41.6809 |
| R-F5-SE | 41.0837 | 11.2373 | 32.9173 | 54.4671 |
| T-S6-N | 24.7597 | 4.8263 | 24.3198 | 11.2104 |
| R-S7-N | 36.087 | 8.9611 | 38.4933 | 20.2074 |

**Table S7.** Pairwise permutational multivariate analysis of variance (PERMANOVA) using distance measures of beta diversity.

| Pairwise comparison | pseudo-F | p-value | q-value |
| --- | --- | --- | --- |
| Cluster 1 vs 2 | 7.101 | 0.009* | 0.009 |
| Cluster 1 vs 3 | 4.369 | 0.021* | 0.021 |
| Cluster 2 vs 3 | 4.457 | 0.101 | 0.101 |

*Statistically significant as p-value < 0.05 (α-value).

**Table S8.** Oxford Nanopore MinION 16S rRNA sequencing and analyses results of sample R-F1-E and S-F3-N. The EPI2ME Fastq16S pipeline was used for the analyses.

| Samples | Sequencing replicate | Unclassified reads | No. of classified genera | No. of classified species |
| --- | --- | --- | --- | --- |
| R-F1-E | Replicate 1 | 964 | 768 | 1835 |
|  | Replicate 2 | 1044 | 794 | 1926 |
| S-F3-N | Replicate 1 | 717 | 714 | 1674 |
|  | Replicate 2 | - | - | - |

No valid reads were obtained with second replicate of sample S-F3-N.
