# Supplementary figures and images for: Exploring taxonomic and functional microbiome of Hawaiian stream and spring irrigation water systems using Illumina and Oxford Nanopore sequencing platforms
Source: Front Microbiol. 2023 Feb 17;14:1039292. doi: 10.3389/fmicb.2023.1039292 (PMC9981659; doi:10.3389/fmicb.2023.1039292)

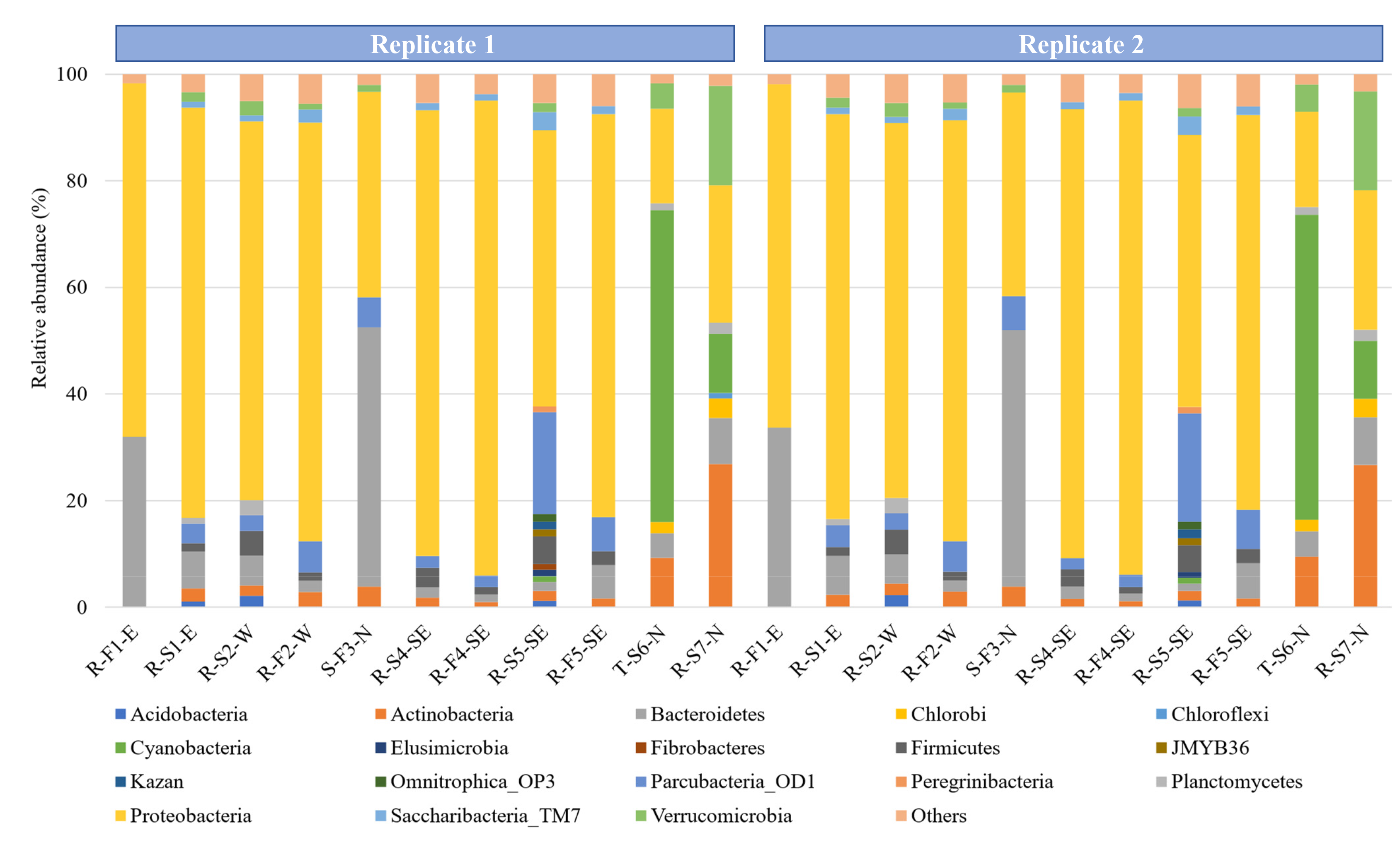

Supplement: Supplementary Figure 1 — Bar plot comparison of phylum level classification, classified with relative abundance of >1% in 11 samples- R-F1-E, R-S1-E, R-S2-W, R-F2-W, S-F3-N, R-S4-SE, R-F4-SE, R-S5-SE, R-F5-E, T-S6-N, and R-S7-N (Replicate 1 and Replicate 2) sequenced for short length amplicon using Illumina iSeq100 and analyzed on EzBioCloud platform. “Others” represents the reads classified with less than <1% relative abundance and remains unclassified in the classification against the database. [file Image_1.TIF]

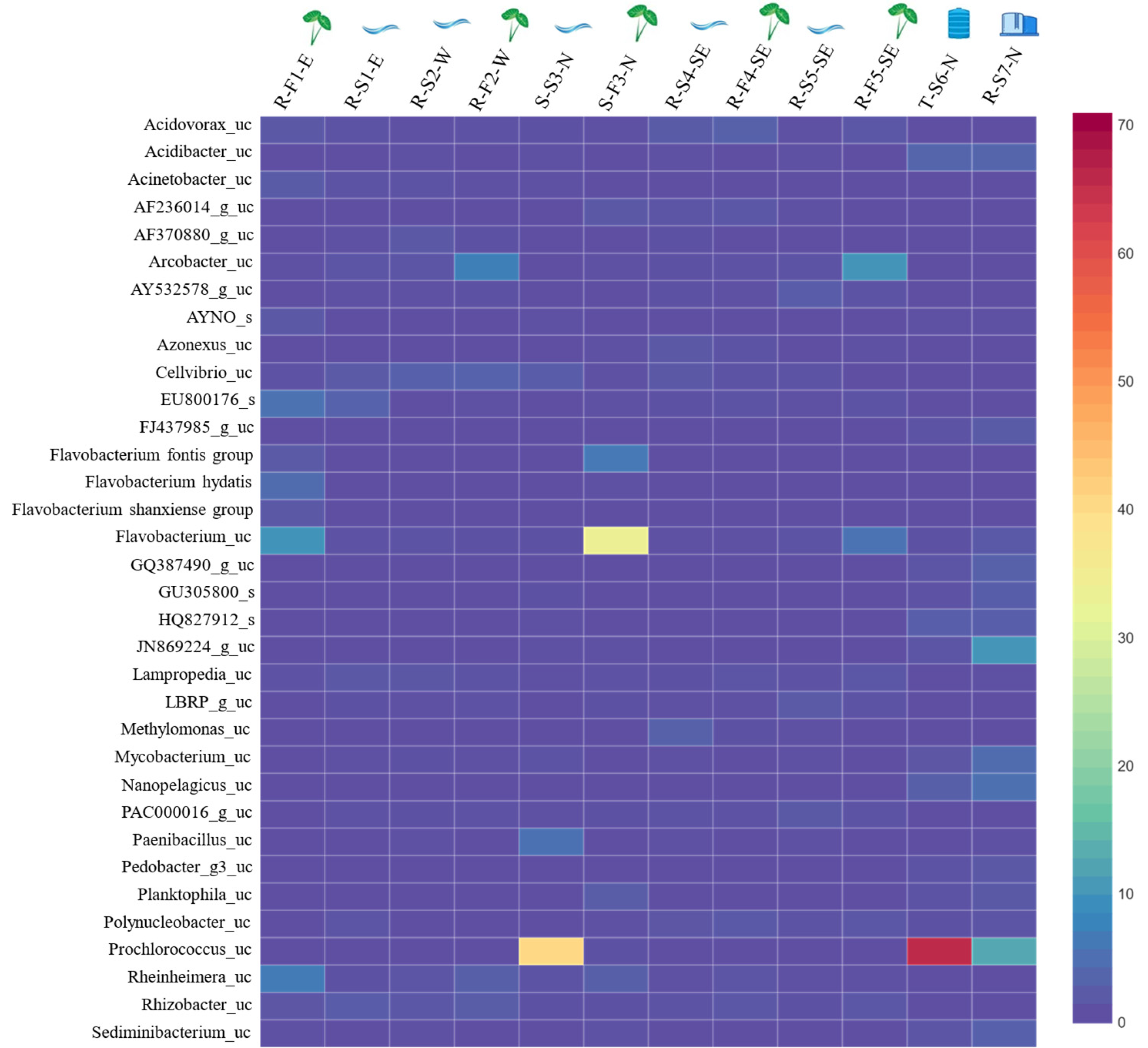

Supplement: Supplementary Figure 2 — Distribution heatmap of bacterial species classified with >1% relative abundance among all the 12 water samples—sequenced for V3-V4 region of 16S rRNA gene region using Illumina iSeq100 sequencing platform. The generated short amplicon reads were analyzed using EzBioCloud platform. The heatmap was generated using display R. [file Image_2.TIF]

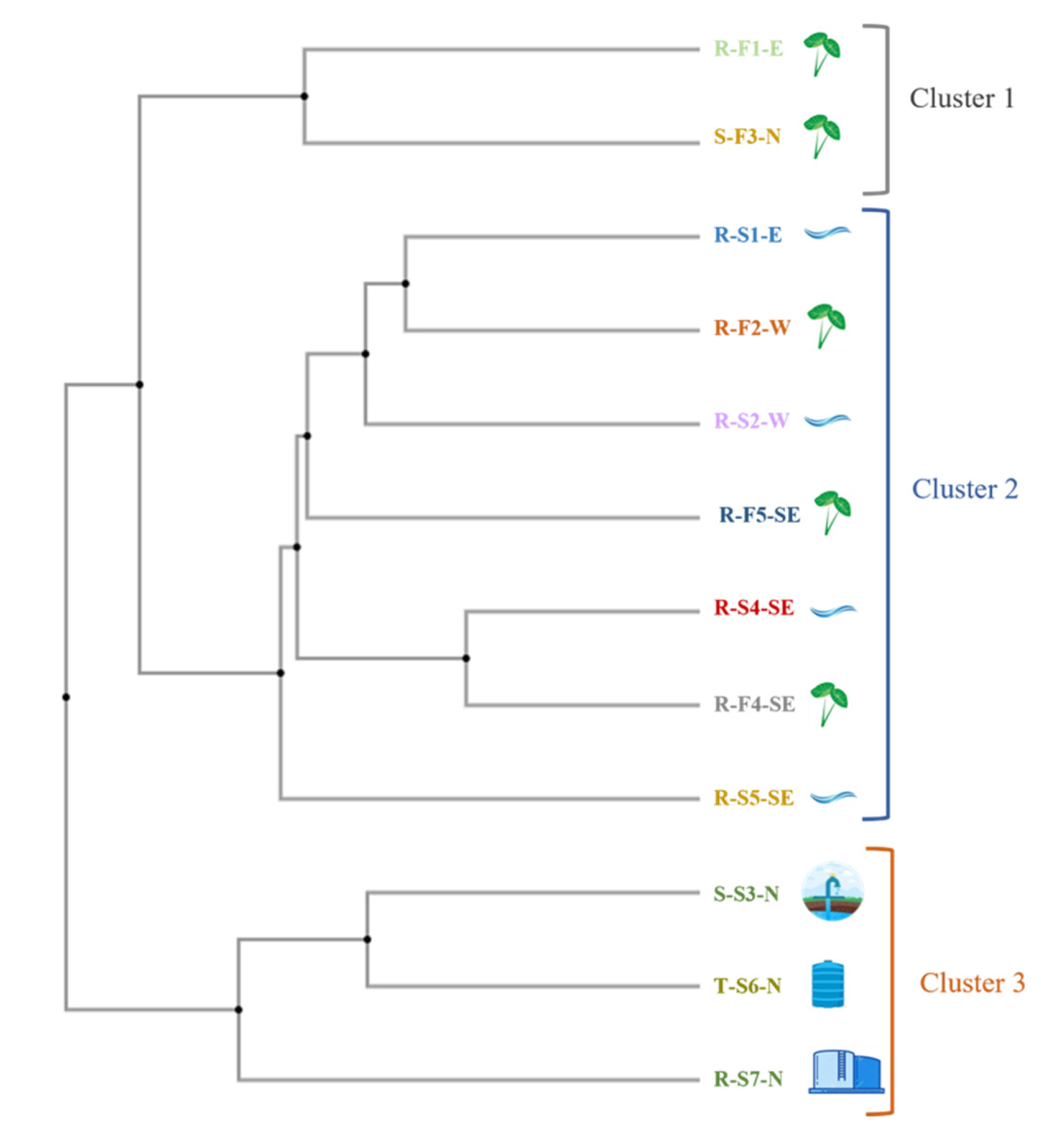

Supplement: Supplementary Figure 3 — UPGMA (unweighted pair group method with arithmetic mean) clustering of water samples based on Bray-Curtis dissimilarity index at genus level. Samples were grouped in three distinctive clusters: Cluster 1 (R-F1-E and S-F3-N) irrespective of water system or geographical location, Cluster 2 (R-S1-E, R-F2-W, R-S2-W, R-F4-SE, R-S4-SE, R-F5-SE, and R-S5-SE) based on irrigation source and associated taro field water, and Cluster 3 (S-S3-N, T-S6-N, and R-S7-N) based on geographical location. [file Image_3.TIF]

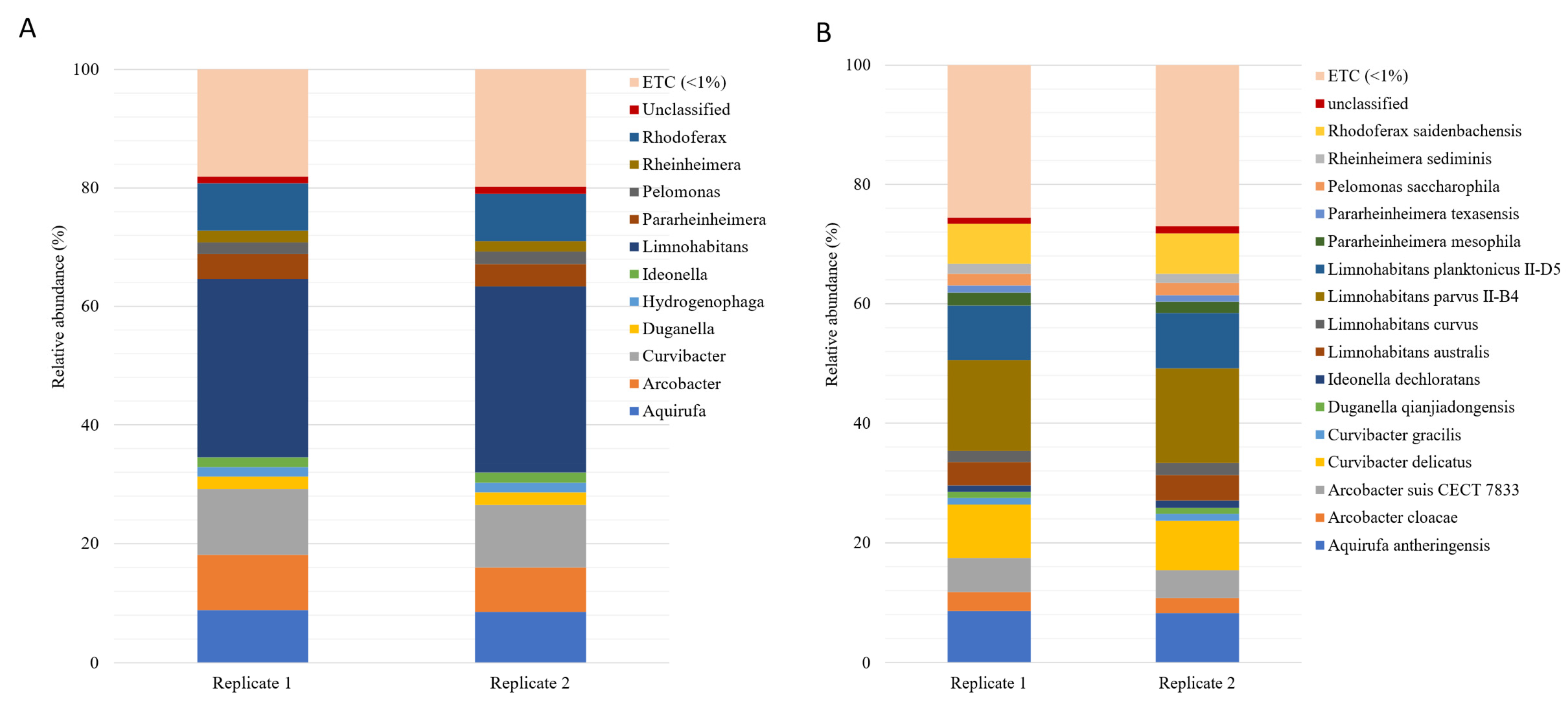

Supplement: Supplementary Figure 4 — Bar plot comparing the (A) genus and (B) species classified with relative abundance of >1% in sample R-F1-E (Replicate 1 and Replicate 2) sequenced for full length amplicon using Oxford Nanopore MinION and analyzed on EPI2ME platform. Input valid reads that were not classified to genus and species levels are represented as “Unclassified”, while “ETC (<1%)” represents the bacterial population identified with relative abundance of <1%. [file Image_4.TIF]

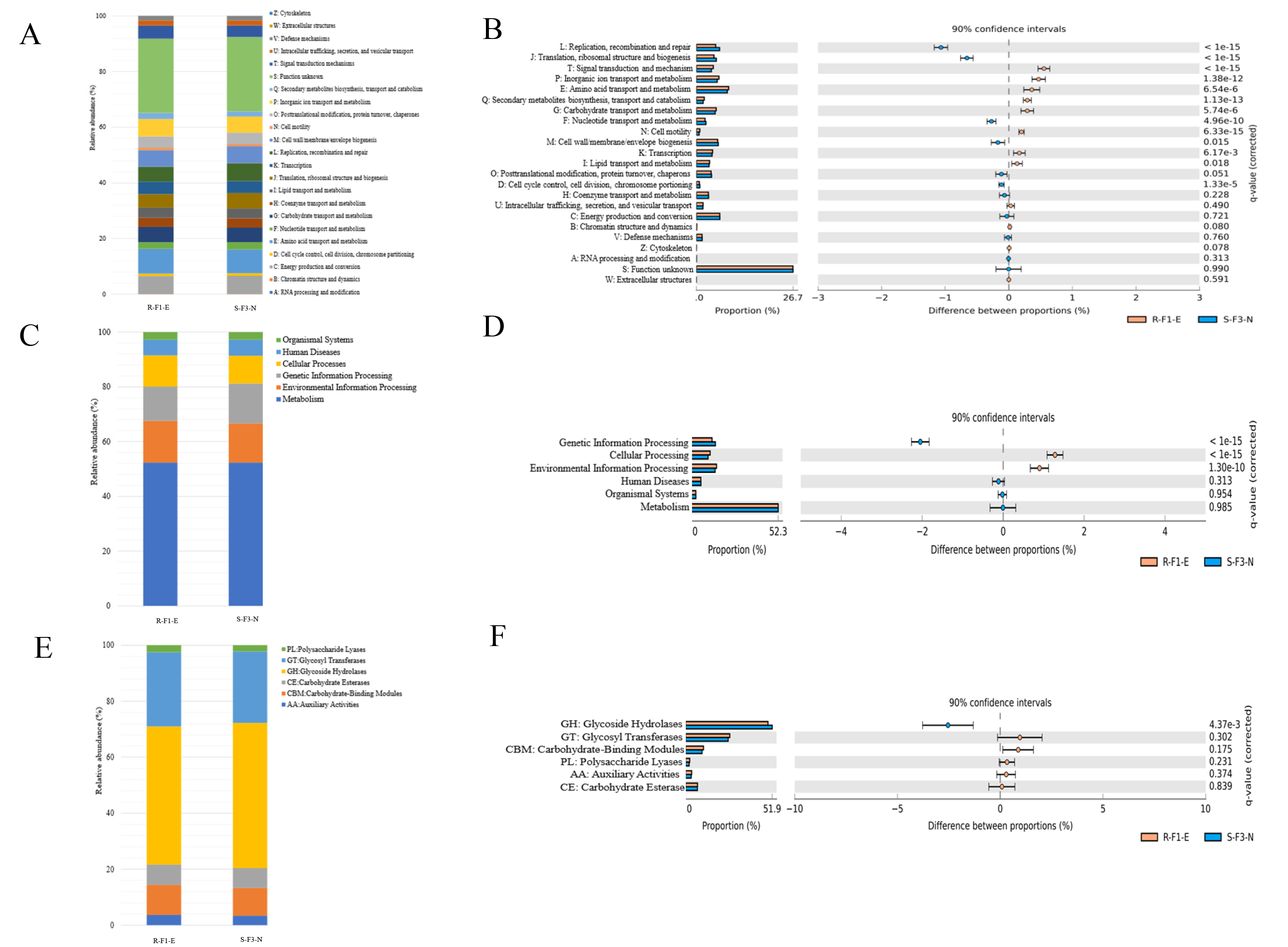

Supplement: Supplementary Figure 5 — Comparison of samples R-F1-E and S-F3-N for relative abundance and statistical differences of annotated gene function profiles based on mapping of assembled metagenomic protein coding sequences to three databases: (A,B) non-supervised Orthologous groups (eggNOG), (C,D) Kyoto Encyclopedia of Genes and Genomes (KEGG), and (E,F) Carbohydrate-Active Enzymes Database (CAZy). Statistic al analyses performed using STAMP v 2.1.3 software, employing Fisher’s exact test with Newcombe-Wilson CI method and Benjamini-Hochberg FDR correction factors, and visualized using extended error bar plots. [file Image_5.TIF]

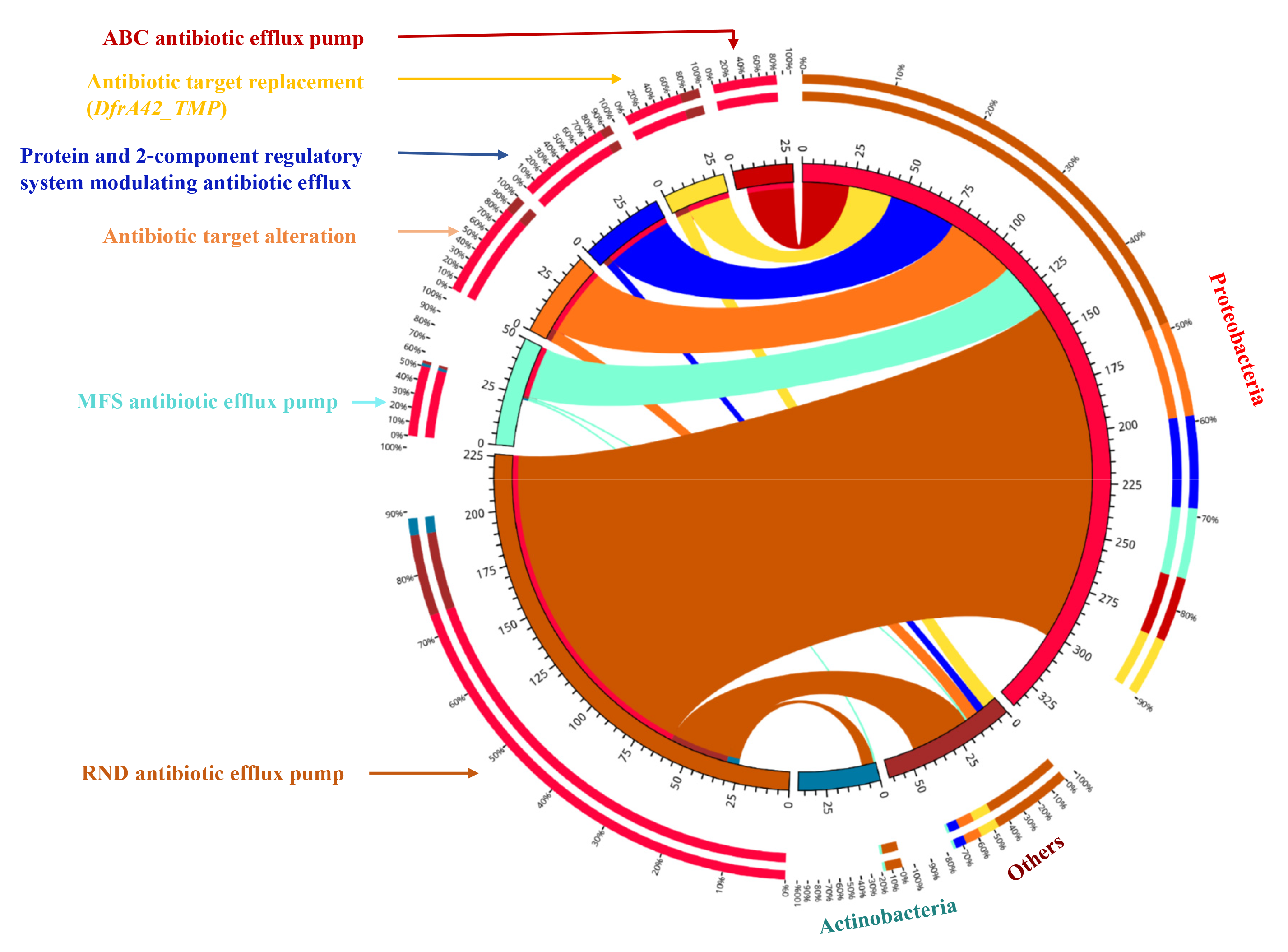

Supplement: Supplementary Figure 6 — Circos analysis displays the corresponding abundance relationship between identified dominant phyla (Proteobacteria and Actinobacteria) along with “other” representation of identified phyla and associated resistance mechanism. Circle chart is divided into two parts. The right side of the circle is phyla information, and the left side of the circle is antibiotic resistance mechanisms. Inner circle with different colors represents different antibiotic resistance mechanisms. The scale represents the relative abundance, and the unit is ppm. The left part represents the sum of relative abundance of different phyla for resistance mechanisms, while the outer right circle vice versa. [file Image_6.TIF]
